# Severe Influenza Virus and Respiratory Syncytial Virus Infections in Intensive Care Over the Last 15 Years

**DOI:** 10.7759/cureus.46966

**Published:** 2023-10-13

**Authors:** Inês B Rua, João Diogo, Gustavo Januário, Rita Moinho, Leonor Carvalho, Patrícia Mação

**Affiliations:** 1 Pediatric Intensive Care Service, Pediatric Hospital, Coimbra Hospital and University Centre, Coimbra, PRT; 2 Department of Pediatrics, Faculty of Medicine, University of Coimbra, Coimbra, PRT; 3 Pediatric Ambulatory Service, Pediatric Hospital, Coimbra Hospital and University Centre, Coimbra, PRT; 4 Pediatric Emergency Service, Pediatric Hospital, Coimbra Hospital and University Centre, Coimbra, PRT

**Keywords:** infectious diseases, rsv, intensive care, pediatrics, influenza

## Abstract

Introduction: Influenza virus is a common agent of pediatric infections. Most cases are mild, but severe illness and death can occur. We aimed to analyze severe cases associated the influenza virus and compare it with respiratory syncytial virus (RSV).

Methods: This is a retrospective study of 0-17-year-old patients admitted to the intensive care unit (ICU) of Hospital Pediátrico, Centro Hospitalar e Universitário de Coimbra (Pediatric Hospital, Coimbra Hospital and University Center), a tertiary pediatric hospital in Coimbra, Portugal, over the last 15 years (2008-2022) due to influenza virus infection. Clinical presentation, severity, and evolution were analyzed. A comparison of children with RSV infection admitted in the same period was performed.

Results: We identified 47 cases of influenza virus infection (34% coinfection with other viruses), median age of 2.3 years (interquartile range (IQR) 6.1), and 38% had comorbidities. The median admissions were three/year (maximum 11 in 2019). Influenza A was identified in 96%. Ninety-six percent had respiratory symptoms, 38% had neurologic symptoms, and 28% had sepsis. The main reason for admission was respiratory failure (68%). The mean pediatric index of mortality 2 (PIM2) at admission was 9±15.9%. Ventilatory support was necessary in 66%, vasoactive support in 19%, and blood products in 17%. The median length of stay was four days (IQR 5). There were four (8.5%) deaths. During the same study period, there were 171 RSV-related admissions. When comparing influenza (group A, without RSV coinfection) and RSV (group B), the first had a higher PIM2 on admission, greater need of ventilatory support, more complications, and higher mortality (p=0.001).

Conclusions: The number of influenza virus infections admitted to ICU was much lower than RSV. However, influenza was more severe and associated with all deaths registered.

## Introduction

Influenza virus is a ribonucleic acid (RNA) virus that belongs to the Orthomyxoviridae family [1]. It is one of the main respiratory pathogens responsible for seasonal epidemic outbreaks in pediatrics [2]. Around 20-30% of children are affected annually [2,3]. The clinical presentation is often benign and is characterized by fever, general malaise, and headache, followed by nasal congestion, non-productive cough, and rhinorrhea [2]. However, in certain cases, influenza may be associated with complications, ranging from acute otitis media to neurological disorders, respiratory failure, and even death [4]. It is estimated that, annually, influenza is responsible for up to 97,200 deaths under the age of five years old, mostly in low-income countries [5].

This infection mainly affects previously healthy children, but complications are more frequent when risk factors, such as asthma, obesity, immunosuppression, and cardiac or neuromuscular diseases, are present, the latter having the highest associated morbidity [2-4,6]. Coinfections with other viruses and secondary bacterial infections are frequently described and seem to play an important role in mortality [6].

Although today there are rapid tests to detect viral antigens in a few minutes, it is recommended that the diagnosis should be confirmed by a more reliable and sensitive test, such as the polymerase chain reaction (PCR) [2]. In most cases, supportive care, namely, with fluid administration, is sufficient. However, in more severe cases, the use of antivirals may be recommended, especially neuraminidase inhibitors [2,7].

Vaccination plays a key role in preventing infections of susceptible children, with the Portuguese Directorate General of Health (DGS) issuing annual recommendations regarding vaccination for risk groups [2,8,9].

Besides influenza, another main agent of pediatric respiratory infections is the respiratory syncytial virus (RSV) [10]. It belongs to the Paramyxoviridae family and is the main cause of lower respiratory infections, namely, acute bronchiolitis and pneumonia, in children under two years of age. It is also the leading cause of pediatric hospitalizations in developed countries [11,12]. RSV is estimated to be responsible for around 101,400 under-five deaths annually, mostly in low-income countries [10].

In this case, treatment involves support therapy [13,14]. A DGS guideline is also available, which identifies patients at risk of more severe diseases, who should receive palivizumab, a monoclonal antibody against RSV, every epidemic season [15]. However, there is currently no specific therapy available [13,14].

We aimed to analyze severe cases of influenza virus infection in children admitted to the pediatric intensive care unit (PICU) and compare them with severe cases of RSV infection.

## Materials and methods

An exploratory study was carried out, with retrospective data collection, of all children and adolescents diagnosed with influenza virus and/or RSV infection, admitted to the PICU of Hospital Pediátrico, Centro Hospitalar e Universitário de Coimbra (Pediatric Hospital, Coimbra Hospital and University Center), a tertiary pediatric hospital in Coimbra, Portugala, over the last 15 years (January 2008 to December 2022).

The identification of cases was carried out using an anonymized database (FileMaker®, Claris International, USA), and in all cases, the diagnosis was confirmed by molecular biology tests (classic PCR or multiple-panel tests) or immunofluorescence.

Demographic data, clinical manifestations, comorbidities, vaccination status concerning influenza vaccine, PIM2 score (Pediatric Index of Mortality) at admission, treatment, and evolution (duration of hospitalization and death) were analyzed. Comorbidities were considered those corresponding to risk groups for severe diseases caused by the influenza identified by the DGS: patients with severe and/or decompensated chronic diseases (pulmonary, including asthma requiring daily inhaled corticosteroids; cardiovascular; renal; hepatic; hematological; neurological and neuromuscular; and metabolic, namely, diabetes mellitus and oncologic conditions) and those who are immunosuppressed and obese [9].

The type of influenza virus and coinfection with other respiratory viruses were also analyzed. For the comparison with cases of RSV infection admitted in the same study period, cases of coinfection with this virus were excluded, and two groups were created: group A (influenza virus infection) and group B (RSV infection). The risk factors for severe diseases in group B were considered those defined by the DGS (prematurity, cyanotic or hemodynamically significant congenital heart disease, chronic lung disease, neuromuscular disease, history of diaphragmatic hernia, pulmonary hypertension, immunodeficiency, or immunosuppression) [15].

Statistical analysis was performed with IBM SPSS Statistics for Windows, version 25 (released 2017; IBM Corp., Armonk, New York, United States). The normality test used was the Kolmogorov-Smirnov test (p=0.05). To analyze the quantitative variables, measures of central tendency and dispersion were used. Qualitative variables were analyzed using absolute and relative frequencies. Differences in distribution between categorical variables were analyzed using Pearson's chi-square test or Fisher's exact test, according to the sample size. Student's t-test was used to compare quantitative variables with normal distribution, and the Mann-Whitney U test was used to compare quantitative variables without normal distribution. The level of statistical significance considered was p<0.05.

## Results

During the study period (2008-2022), 47 children/adolescents with influenza virus infection were admitted to the PICU, corresponding to 0.8% of all admissions. Fifty-seven percent were male, and the age at admission ranged from four days to 16.3 years, with a median of 2.3 years (interquartile range (IQR) 6.1). Two patients (4%) were newborns.

Eighteen cases (38%) had comorbidities, the most frequent being asthma under therapy with inhaled corticosteroids (n=7), congenital heart disease (n=5), and chronic respiratory failure under non-invasive ventilation (NIV) at home (n=3). Among the group of patients with recommendation for influenza vaccination according to the DGS criteria, the vaccination status was only known in 11 cases (61%), and five of these (45%) had been vaccinated in that season.

Coinfection with other respiratory viruses was documented in 16 cases (34%), with RSV being the most frequent: in 11 cases (nine alone and two together with other viruses than influenza). It was possible to identify the type of influenza virus in practically all cases (except one), with type A being identified in 96%. Median admissions were three/year, with a peak of 11 cases in 2019.

Regarding clinical manifestations, 96% had respiratory symptoms, 38% had neurological symptoms, 28% had sepsis, and 11% had gastrointestinal symptoms. Reasons for admission were respiratory failure in 68%, seizures/seizure status in 13%, altered state of consciousness (including coma) in 9%, septic shock in 4%, apnea in 2%, cardiac arrest in 2%, and fever in a newborn in 2%. The average PIM2 on admission was 9±15.9%.

During hospitalization, ventilatory support was required in 66% of the patients (n=31), of which NIV in 15 cases and invasive ventilation (IV) in 21 cases. The median duration of NIV was 72 hours (IQR 95) and that of IV was 72 hours (IQR 120). Nineteen percent also needed vasoactive support, 17% blood products, 17% antiepileptic therapy, and 2% renal replacement therapy. Regarding other therapies, 98% had antibiotics prescribed, oseltamivir was prescribed in 94%, bronchodilators in 36%, and systemic steroids in 30%.

At least one complication was identified in 85% of patients: 74% secondary bacterial infection, of which 23% sepsis, 19% pulmonary atelectasis, and 6% pleural effusion. The median length of stay was four days (IQR 5), with a maximum of 35 days. There were four deaths (8.5%), including two children with comorbidities (a 10-year-old with neuromuscular disease and chronic respiratory failure hospitalized for worsening respiratory failure and a 15-month-old with trisomy 21 and heart disease hospitalized for cardiorespiratory arrest hospitalization and cardiogenic shock) and two without risk factors for severe influenza, namely, a five-year-old with severe acute respiratory distress syndrome, sepsis, and pneumococcal pneumonia with pleural effusion with fulminant evolution and a four-year-old who presented with a seizure status in the context of acute necrotizing encephalitis, which progressed to brain death. Three patients (6%) had sequelae, including neurological (n=2) and finger amputation (n=1).

**Figure 1:**
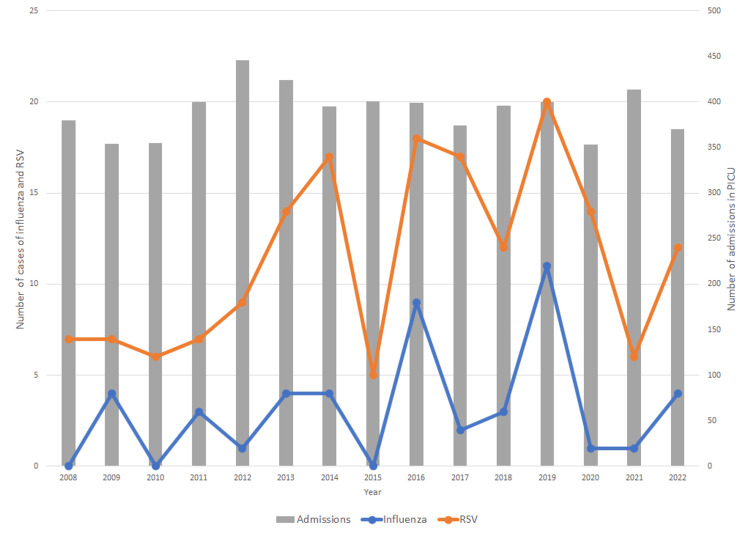
Annual distribution of admissions and cases admitted to the PICU with influenza virus and RSV infection from 2008 to 2022 (n=171). PICU: pediatric intensive care unit; RSV: respiratory syncytial virus

For the comparison with cases of RSV infection admitted in the same study period, cases of coinfection with this virus were excluded (n=11), and two groups were created: group A (influenza virus infection; n=36) and group B (RSV infection; n=171). Regarding the group with RSV infection, 59% were male. Age at admission varied between eight days and five years, with a median of one month old (IQR 0.2). About 33% were newborns and 95% were less than two years old. Twenty-six percent had at least one risk factor for severe infections, including prematurity (22%), congenital heart disease (2%), and neuromuscular disease (2%). RSV appeared in a coinfection with other viruses in 16% of cases, mostly rhino/enterovirus (7%) and adenovirus (6%). The median number of admissions per year was 12, with a peak of 20 cases in 2019. The distribution of admissions per year is shown in Figure 1.

Regarding the clinical presentation, 100% had respiratory symptoms, 10% sepsis, 8% neurological symptoms, and 5% gastrointestinal symptoms. The reasons for admission were respiratory failure in 80%, isolated apnea in 15%, shock in 2%, seizures/seizure status in 1%, cardiac arrest in 1%, and severe hyponatremia in 1%. The average PIM2 at admission was 1.7±4%.

Seventy-two percent of cases were medicated with antibiotics, 36% with bronchodilators, and 26% with systemic steroids. The median length of stay was five days (IQR 4). There were no deaths and no patient had sequelae. Other characteristics, namely, treatment options, are described in Table 1.

**Table 1:** Demographic and clinical characteristics of the influenza virus (group A; n=36) and RSV (group B; n=171) infection cases admitted in the PICU between 2008 and 2022 IQR: interquartile range; n: sample size; PIM2: Pediatric Index of Mortality score; SD: standard deviation; * Fisher's exact test; # Mann-Whitney U test

	Group A: Influenza virus (n=36)	Group B: RSV (n=171)	p-value
Age (years) median (IQR)	3,4 (7,8)	0,1 (0,2)	<0,001^#^
Male (n, %)	19 (52,8%)	100 (58,5%)	0,529*
Comorbidities (n, %)	14 (38,9%)	44 (25,7%)	0,11*
Influenza vaccination (n, %)	2 (7,4%)	Not-applicable	
PIM2 (mean ± SD)	10,9 ±17,7	1,7 ± 4	<0,001^#^
Invasive and non-invasive ventilation (n, %)	24 (66,7%)	73 (42,7%)	0,011*
Non-invasive ventilation (n, %)	13 (36%)	49 (28,7%)	
Invasive ventilation (n, %)	16 (44%)	38 (22,2%)	
Non-invasive ventilation only (n, %)	8 (22,2%)	35 (20,5%)	
Vasoactive support (n, %)	8 (22,2%)	22 (12,9%)	0,147*
Blood products (n, %)	8 (22,2%)	6 (3,5%)	0,001*
Renal replacement therapy (n, %)	1 (2,8%)	0 (0%)	0,174*
Complications (n, %)	31 (86,1%)	114 (66,7%)	0,008*
Length of stay (days), median (IQR)	4,5 (7)	5 (4)	0,212^#^
Sequelae (n, %)	3 (8,3%)	0 (0%)	<0,001*
Mortality (n, %)	4 (11,1%)	0 (0%)	0,001*

Comparing the two groups, it was found that the median age was significantly lower in group B (VSR; 3.4 vs 0.1 years, p<0.001). Overall, more than one-third of the patients had comorbidities for severe diseases, with no statistically significant difference between the two groups. Children in group A (influenza infection) had a higher PIM2 severity score on admission, greater ventilatory support, more complications, and higher mortality (11.1% vs 0%, p=0.001). A comparative analysis between both groups can also be found in Table 1.

## Discussion

Our study analyzed data, from the last 15 years, of children hospitalized with severe influenza in a PICU of a tertiary pediatric hospital in Portugal. Nowadays, respiratory viruses are increasingly recognized as potentially serious pathogens, and this study described and compared the cases and severity of influenza and RSV infections. There has been an upward trend in the number of hospitalizations due to severe influenza in recent years.

The majority of hospitalized patients were male, similar to what was identified in another study [16]. We also found a median age of 2.3 years in the studied population, with the age group under five years of age being the most prevalent. This age group was already recognized as one of the most frequently affected and at risk for severe influenza illness [16,17].

In our sample, approximately 38% of the patients had comorbidities, which was lower than the proportion reported in other series in which comorbidities were present in approximately 80% of the cases [16,17]. It is noteworthy, however, that, as in our sample, about half of the deaths from influenza occur in children without risk factors for severe illnesses [18].

Only five cases (45%) of the total number of patients with recommendation for influenza vaccination, according to DGS rules, were vaccinated during the corresponding season. In Portugal, as in other European countries, vaccination is recommended annually for children over six months that are considered a risk group [9]. Reasons for non-vaccination were not analyzed in our study. We should emphasize, however, that vaccination is recognized as one of the most effective means of preventing influenza infection and complications during epidemics.

Type A influenza virus was the most frequently identified type of virus, being present in 96% of cases. Despite the recent literature, namely, comparative studies between type A and type B influenza infections, apparently demonstrating a similar severity in pediatric age, our series shows an almost absence of type B influenza as a cause of severe disease (only two cases) [19,20]. Some studies relate the potential severity with the different subtypes of type A influenza, with greater severity being recognized in periods with increased circulation of the AH3N2 subtype, but in our sample, the virus subtype was not analyzed [21].

As expected, respiratory manifestations were the most frequent in the studied sample of children with severe influenza. However, we highlight the high proportion of cases with neurological symptoms (38%). Most studies refer to neurological manifestations in up to 26% of children hospitalized with severe influenza infection [22-25]. The presence of these neurological manifestations is usually associated with poor prognosis and even death [22]. In our series, we had one death due to encephalitis and two cases with neurological sequelae.

Antibiotics were prescribed practically in all cases (98%). We consider the prescription rate to be high, which could be justified by the suspicion of secondary bacterial infection, although this was identified as a complication in only 74% of cases [17].

Oseltamivir, a neuraminidase inhibitor, was prescribed in 94% of cases, although it is recommended for the treatment of all infections requiring hospitalization due to influenza. Its greatest effect is obtained when it is administered within the first 48 hours of disease, decreasing with late administration, which may have justified its non-use in specific cases [26,27].

Comparison of influenza and RSV

Cases of influenza and RSV infections requiring admission to the PICU had a similar trend over the years (Figure 1). The age of the patients was significantly higher in the group with influenza virus infection, which can be explained by the predilection of RSV for the first months of life, where it is known to be more severe, with a high number of newborns in our sample (33%) [28]. The greater severity of influenza is evident in the comparison between the two groups, namely, with a higher mean of the PIM2 score (admission severity index), a higher percentage of ventilatory support, use of blood products, and number of complications.

The existence of sequelae and mortality was exclusive to the influenza group, although both groups had a similar percentage of comorbidities [17]. As described in the literature and as verified in our sample, severe cases and mortality also occurred in previously healthy children. Although mortality associated with influenza is not frequent, it occurred in 8.5% of our study, similar to what was already known [3,16,17].

Despite being a retrospective study, which constitutes a limitation in the study design, the approach to these patients and their clinical records were almost always complete, since our PICU is an audited and certified service with a reduced clinical team, uniform in their attitudes. Although there are studies that compared the severity of influenza with other viruses [17], our study seems to be a pioneer in the comparative analysis of influenza and RSV in the central region of the country, being also one of the most recent.

## Conclusions

Most influenza and RSV infections have a benign and self-limited course, but the potential for severity exists and is not just associated with the existence of previous comorbidities, as both viruses can cause severe diseases, even in previously healthy children.

In this study, a population of pediatric patients with severe influenza and RSV infections requiring hospitalization in the only service that provides intensive care in the central region of Portugal was characterized. Influenza virus was, compared to RSV, associated with a higher PIM2 score on admission, a greater need for invasive and non-invasive ventilation, a greater need for vasoactive support and the use of blood products, and a greater number of complications and was ultimately responsible for all recorded deaths and sequelae.
